# A signature-based classification of lung adenocarcinoma that stratifies tumor immunity

**DOI:** 10.3389/fonc.2022.1023833

**Published:** 2023-01-12

**Authors:** Xun Zhang, Dizhi Jiang, Shunjia Li, Xinyu Zhang, Wendi Zheng, Bo Cheng

**Affiliations:** Qilu Hospital of Shandong University, Cheeloo College of Medicine, Shandong University, Jinan, China

**Keywords:** lung adenocarcinoma, immunophenotypes, tumor-infiltrating immune cells, prognostic model, immunotherapy

## Abstract

**Background:**

Immune-related subgroup classification in immune checkpoint blockade (ICB) therapy is largely inconclusive in lung adenocarcinoma (LUAD).

**Materials and methods:**

First, the single-sample Gene Set Enrichment Analysis (ssGSEA) and K-means algorithms were used to identify immune-based subtypes for the LUAD cohort based on the immunogenomic profiling of 29 immune signatures from The Cancer Genome Atlas (TCGA) database (n = 504). Second, we examined the prognostic and predictive value of immune-based subtypes using bioinformatics analysis. Survival analysis and additional COX proportional hazards regression analysis were conducted for LUAD. Then, the immune score, tumor-infiltrating immune cells (TIICs), and immune checkpoint expression of the three subtypes were analyzed. The Gene Ontology (GO) and Kyoto Encyclopedia of Genes and Genomes (KEGG) of the differentially expressed genes (DEGs) between three immune-based subtypes were subsequently analyzed for functional enrichment pathways.

**Result:**

A total of three immune-based subtypes with distinct immune signatures have been identified for LUAD and designated as cluster 1 (C1), cluster 2 (C2), and cluster 3 (C3). Patients in C3 had higher stromal, immune, and ESTIMATE scores, whereas those in C1 had the opposite. Patients in C1 had an enrichment of macrophages M0 and activation of dendritic cells, whereas tumors in C3 had an enrichment of CD8^+^ T cells, activation of CD4^+^ memory T cells, and macrophages M1. C3 had a higher immune cell infiltration and a better survival prognosis than other subtypes. Furthermore, patients in C3 had higher expression levels of immune checkpoint proteins such as PD-L1, PD1, CTLA4, LAG3, IDO1, and HAVCR2. No significant differences were found in cluster TMB scores. We also found that immune-related pathways were enriched in C3.

**Conclusion:**

LUAD subtypes based on immune signatures may aid in the development of novel treatment strategies for LUAD.

## 1 Introduction

Lung cancer is the leading cause of cancer-related mortality and the most frequently diagnosed cancer worldwide (1–5). Non-small cell lung cancers (NSCLCs) account for approximately 85% of all lung cancers, with lung squamous cell carcinomas (LUSCs) and lung adenocarcinomas (LUADs) being the most common (6). LUAD is the most prevalent primary lung tumor, accounting for 40% of all primary lung tumors (7). Despite the striking clinical improvements, the 5-year survival rate for LUAD patients is only 18% (8).

Blocking immune checkpoints is one of the most promising strategies for boosting anti-tumor immunity (9–13). Immunotherapy (IO) has revolutionized lung cancer treatment, significantly improving overall survival (OS) (14–17). Immune checkpoints, such as PD-L1 (18–20), CTLA4 (21, 22), TIGIT (23), and LAG3 (24), have emerged as promising drug targets for cancer IO. In recent years, IO has gained widespread acceptance as the first-line treatment for non-oncogene-driven lung cancer (25, 26). However, a significant proportion of patients experience immune resistance and disease progression. Although various putative biomarkers have been investigated for predicting response to immune checkpoint inhibitors (ICI), there is currently no common biomarker available for patients receiving ICI. Many researchers believe that IO based solely on a limited number of immune checkpoints is insufficient and that physicians should consider immune characteristics in a broader sense (including tumor-infiltrating lymphocytes and gene signatures) when formulating treatment plans.

Several studies have been conducted on the immune characteristics of various solid tumor types. Daniela et al. identified the immune class of hepatocellular carcinoma as patients with a high degree of immune infiltration and molecular features (27). The immune-based assay “Immunoscore” was developed to quantify *in-situ* T cells in colon cancer patients and outperformed the TNM stage (28). According to evidence from other cancer types, ICI therapy can benefit patients with an inherently inflamed tumor microenvironment (TME), whereas immune-excluded tumors are prone to resistance (29). Although some studies have focused on lung cancer with typical immune characteristics, models based on immune characteristics to help find patients best suited for immunotherapy are still lacking.

Classification of tumors as “hot,” “cold,” or “altered” based on CD3^+^ and CD8^+^ T cell infiltration (30), PD-L1 expression, and tumor mutation burden (TMB) at the center and margin of the tumor is an essential determinant of IO response in solid cancers (31, 32). While hot and cold tumor phenotypes have been shown to correlate with treatment response and overall cancer outcome, specific immune cell subsets, including regulatory T cells (Tregs), myeloid-derived suppressor cells (MDSCs), and tumor-associated macrophages (TAMs), have been shown to alter this relationship (33).

Using the Nonnegative Matrix Factorization (NMF) method, we identified LUAD subtypes based on 29 immune signatures. An immune-classification method for LUAD patients is being developed to identify those who respond to anti-PD-1/PD-L1 therapy.

## 2 Material and methods

### 2.1 Data source

The data for LUAD, including RNA expression, somatic mutation, and clinical pathological information, was obtained from TCGA database (https://tcga-data.nci.nih.gov/tcga/).

### 2.2 Immune signature-based subtype classification

The number of clusters across the tumor samples was determined using a consensus cluster algorithm. Non-negative matrix factorization (NMF) is an effective method for reducing data dimension and has a wide range of applications for identifying the functional components of multidimensional complex data. The NMF method in the R software ‘CancerSubtypes’ package was utilized to stratify tumor immunity based on TCGA data (34). For subtype clustering, 29 immune signatures, including immune cell types, functions, and pathways, were utilized (**Supplementary Table 1**). Gene set variation analysis (GSVA) is a nonparametric, unsupervised analytical method used primarily to evaluate the sequencing gene set enrichment results (35, 36). From a bioinformatics standpoint, the goal is to define phenotypic differences. In our study, we quantified immune signatures using the R package “GSVA” and the ssGSEA algorithm (37). Based on ssGSEA scores for immune signature enrichment, all LUAD were hierarchically clustered into three subtypes: cluster 1 (C1), cluster 2 (C2), and cluster 3 (C3).

### 2.3 Survival analysis

Survival analysis is a method for evaluating and deducing the survival times of patients with different subtypes of LUAD based on clinical data obtained from TCGA. The survival analysis for our study was carried out by utilizing the survival package included in the R software. Kaplan–Meier curves and the Logrank test were used to compare the differences in survival rates between patients with different types of LUAD.

### 2.4 Multivariate Cox regression analyses

We investigated the prognostic impact of clusters and clinical factors, such as age, gender, pathologic stage, race and TNM stage (AJCC, American Joint Committee on Cancer/UICC, Union internationale cancer control, 8th edition). Multivariate Cox regressions were conducted to eliminate confounding factors, and the “forest-plot” R package was used to display each variable’s *P* value, HR, and 95% CI. Principal component analysis (PCA) was conducted using the “prcomp” feature of the “stats” package in R.

### 2.5 Immunogenomic features of LUAD

Using expression data, the Estimation of STromal and Immune cells in MAlignant Tumor tissues using Expression data (ESTIMATE) algorithm calculated immune and stromal scores to quantify the infiltration of immune and stromal cells and evaluate the immune activity of the tumor. ESTIMATE can generate immune scores (positively reflecting the abundance of immune cells), stromal scores (positively reflecting the abundance), and ESTIMATE scores (positively reflecting the non-tumor component).

CIBERSORT (37) was used to calculate the relative frequencies of 22 different types of TIICs in each tumor tissue, namely: dendritic cells resting, dendritic cells activated, monocytes, mast cells resting, mast cells activated, neutrophils, plasma cells, eosinophils, B cell naive, B cell memory, T cells regulatory (Tregs), T cells CD4^+^ naive, T cells CD4^+^ memory resting, T cells CD4^+^ memory activated, T cells follicular helper, T cells gamma delta, T cells CD8^+^, NK cells resting, NK cells activated, macrophages M0, macrophages M1, and macrophages M2. *P* < 0.05 and 1000 permutations were used as sample deconvolution criteria. The Kruskal–Wallis test was used to calculate the proportion of TIICs and immune checkpoint protein expression in three immune-based cell subpopulations.

### 2.6 Identification of mutational patterns and TMB

The prespecified definition of TMB-high status was at least 10 mutations per megabase. TMB has been retrospectively correlated with response to immune checkpoint blockade. VarScan was used to analyze MAF files for somatic variants in each LUAD sample, and the maftools package generated waterfall plots visualizing the somatic variants of samples in C1 and C3 (38). Then, after sorting the mutation frequency in TCGA database from highest to lowest in immune-based subtypes, we selected the top 30 mutated genes in C1 and C3.

The data from whole-exome sequencing (WES) were analyzed to calculate the TMB score for LUAD samples. The total number of non-synonymous somatic variants was classified by the exome size for the TMB score of the TCGA database (39). In addition, we also analyzed the TMB score between C1 and C3 subtypes using the student’s t-test.

### 2.7 Gene functional enrichment analysis

Differentially expressed genes (DEGs) in C1 and C3 were identified using the R limma package. DEGs were identified as genes with |Log (Fold change)| >1.5 and false discovery rate (FDR) < 0.05. Furthermore, protein-protein interaction (PPI) network was constructed using the STRING database (https://string-db.org/). Cystoscope 3.7.2 (https://cytoscape.org/) was used to examine network topology. Hub genes are those with a degree of 10 or more. Gene Ontology (GO) and Kyoto Encyclopedia of Genes and Genomes (KEGG) pathway enrichment analyses were carried out to evaluate the pathway enrichment in DEGs.

### 2.8 Statistical analysis

The Chi-square test was used to compare categorical variables. The student’s t-test was used to compare continuous variables between two groups. The one-way analysis of variance (40) or Kruskal–Wallis tests were used to compare continuous variables between three groups. Kaplan–Meier curves and Logrank tests were used to compare survival differences between immune-based subgroups of patients. Univariate and multivariate Cox regression analyses were conducted to eliminate the confounding factors. Linear regressions were used for simple correlation. All analyses were performed using R version 4.2.5 (https://www.r-project.org/). *, *P* < 0.05; **, *P* < 0.01; ***, *P* < 0.001.

## 3 Results

### 3.1 Identification of immune-based subtypes of LUAD

**Figure 1:**
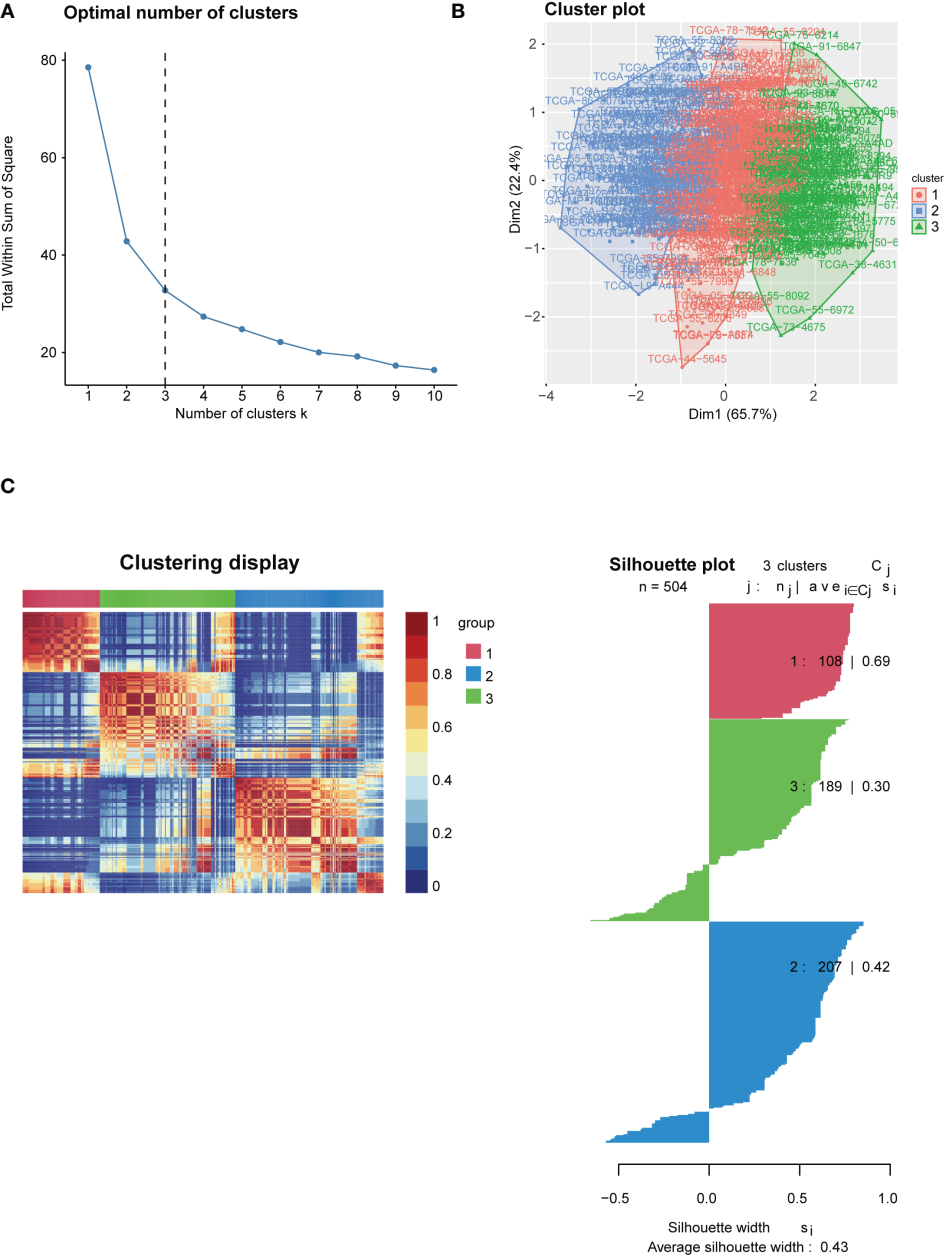
Identification of immune-based subtypes of LUAD. **(A)** Identification of best cutoff of the cluster; **(B)** Distribution of each cluster by the PCA method in the factoextra package; **(C)** Visualization of each cluster using the NMF method. Identification of the value of grouping by silhouette width plots.

To identify the immune signatures among LUAD, LUAD sequencing data were screened and downloaded from TCGA (https://tcga-data.nci.nih.gov/tcga/; 504 cancer cases). The hallmarks of 29 immune signatures (gene sets) were subsequently downloaded from GSVA. The specific immune signature was shown in **Supplementary Table 1**. Immune signature score was quantified by the single sample Gene Set Enrichment Analysis (ssGSEA) algorithm using the R package “gsva” (http://www.bioconductor.org/packages/release/bioc/html/GSVA.html). Using the NMF method, K=3 was determined to be the optimal number of clusters, indicating that LUAD samples could be divided into three subtypes based on the GSVA immune-based signature score (**Figure 1A**). The three subdivided groups are displayed in **Figure 1B**. The silhouette width plot and cluster display plot demonstrated subtype identification for credibility (0.43 average silhouette width, **Figure 1C**).

### 3.2 Correlation between LUAD subtypes and clinical characteristics

**Figure 2:**
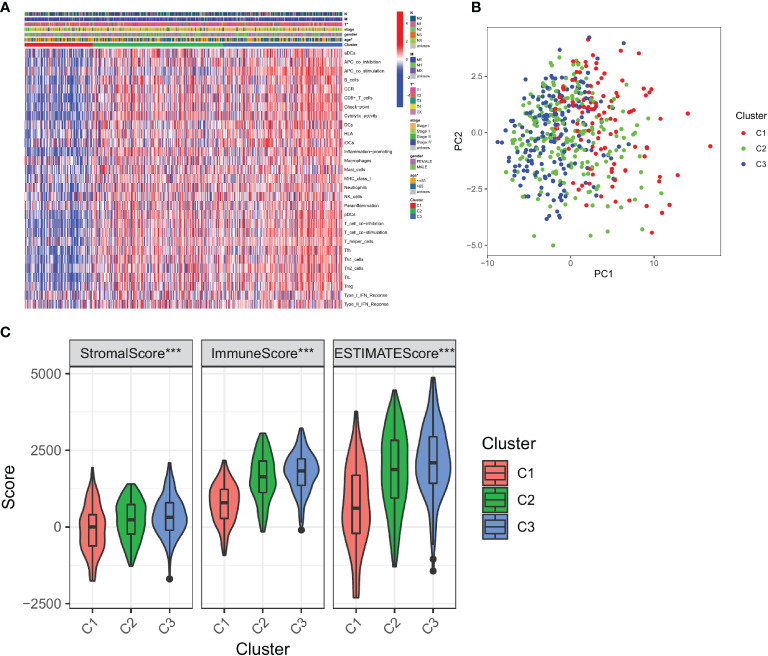
Correlation between immune-based subtypes of LUAD and clinical characteristics. **(A)** Correlation between the expression of 29 immune signature genes and clinical characteristics in three clusters; **(B)** PCA analysis of three clusters; **(C)** Immune infiltration assessment in three clusters. *, *P* < 0.05 and ***, *P* < 0.001.

To test the clinical significance of 29 immune signatures, we examined the relationship between clinical characteristics and subtypes. The differently 29 immune signatures scores with significance by pairwise comparison were enriched and calculated in each cluster (adjusted *P* value <0.05). A total of 29 significant immune signatures were enriched as common differently expressed immune signatures in the 3 subtypes. The C1 and C3 subtypes showed significant differences in 29 immune signature scores among the three subtypes. The correlation between clinical characteristics and immune-based subtypes was determined in the TCGA-LUAD cohort. The proportion of patients over 65 years (55.56%) in C3 was significantly higher than that of patients over 65 years or younger (40.74%). However, the proportion of patients over 65 years (39.81%) in C1 was significantly lower than that of patients aged 65 years or younger (59.26%). Furthermore, T stages of patients in C3 were concentrated in T1 and T2 stage, while the proportion of T3 and T4 stages of patients in C3 significantly lower than the figure for C1. But patients in C2 frequently share a moderate T stage with statistical significance (**Figure 2A**). In addition, the PCA result revealed that patients could be accurately classified using 29 immune signature scores, especially C1 and C3 (**Figure 2B**). What’s more, **Table 1** outlined the clinical significance of the three immune-based subtypes. The patient’s age and T stage varied significantly among the three immune-based subtypes (*P <*0.05). Then TCGA datasets were analyzed to determine the statistical difference in ESTIMATE scores between C1 and C3. There were significant differences among the three subtypes in Stromal Score, Immune Score and ESTIMATE Score (all *P* < 0.001; **Figure 2C**). These results indicate that, among these clinical characteristics, patients in C1 tend to be in the poorest condition, while those in C3 tend to be in the best condition.

**Table 1:** The relevance among subtypes and clinical data inTCGA-LUAD.

Characteristic	Total	C1	C2	C3	*P* value
Age, n (%)					
<=65	238 (47.22%)	64 (59.26%)	97 (46.86%)	77 (40.74%)	0.0152
>65	256 (50.79%)	43 (39.81%)	108 (52.17%)	105 (55.56%)	
unknown	10 (1.98%)	1 (0.93%)	2 (0.97%)	7 (3.7%)	
Gender, n (%)					
Female	270 (53.57%)	59 (54.63%)	110 (53.14%)	101 (53.44%)	0.9678
Male	234 (46.43%)	49 (45.37%)	97 (46.86%)	88 (46.56%)	
Pathologic stage,n (%)					
Stage I	270 (53.57%)	49 (45.37%)	117 (56.52%)	104 (55.03%)	0.3197
Stage II	119 (23.61%)	26 (24.07%)	46 (22.22%)	47 (24.87%)	
Stage III	81 (16.07%)	25 (23.15%)	29 (14.01%)	27 (14.29%)	
Stage IV	26 (5.16%)	7 (6.48%)	11 (5.31%)	8 (4.23%)	
unknown	8 (1.59%)	1 (0.93%)	4 (1.93%)	3 (1.59%)	
T stage, n (%)					
T1	168 (33.33%)	23 (21.3%)	82 (39.61%)	63 (33.33%)	0.042
T2	269 (53.37%)	65 (60.19%)	101 (48.79%)	103 (54.5%)	
T3	45 (8.93%)	11 (10.19%)	17 (8.21%)	17 (8.99%)	
T4	19 (3.77%)	8 (7.41%)	5 (2.42%)	6 (3.17%)	
TX	3 (0.6%)	1 (0.93%)	2 (0.97%)	0 (0%)	
N stage, n (%)					
N0	325 (64.48%)	63 (58.33%)	137 (66.18%)	125 (66.14%)	0.6048
N1	94 (18.65%)	21 (19.44%)	37 (17.87%)	36 (19.05%)	
N2	71 (14.09%)	21 (19.44%)	28 (13.53%)	22 (11.64%)	
N3	2 (0.4%)	0 (0%)	1 (0.48%)	1 (0.53%)	
unknown	12 (2.38%)	3 (2.78%)	4 (1.93%)	5 (2.65%)	
M stage, n (%)					
M0	335 (66.47%)	81 (75%)	128 (61.84%)	126 (66.67%)	0.8191
M1	25 (4.96%)	6 (5.56%)	11 (5.31%)	8 (4.23%)	
unknown	144 (28.57%)	21 (19.44%)	68 (32.85%)	55 (29.1%)	
Race, n (%)					
Caucasians	387 (76.79%)	79 (20.41%)	147 (37.98%)	161(41.60%)	0.001
Asian	7 (1.39%)	6 (85.71%)	0	1 (14.29%)	
Other	110 (21.82%)	23 (20.91%)	42 (38.18%)	45 (40.91%)	

### 3.3 Survival outcomes of immune signature-based subtypes

**Figure 3:**
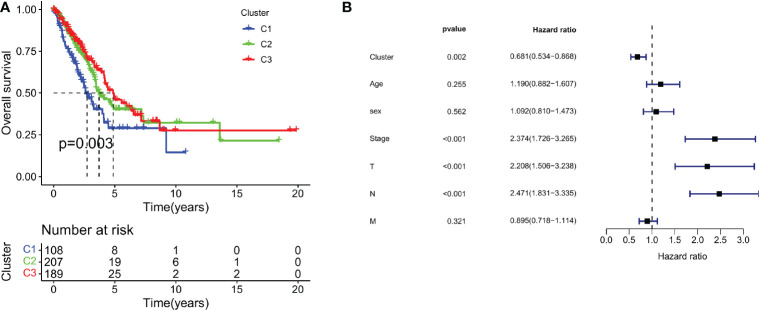
Survival outcomes of immune signature-based subtypes. **(A)** Survival analysis of three clusters; **(B)** Multivariate Cox regression analysis of clusters and clinical characteristics.

To test the survival significance of 29 immune signatures, we examined the relationship between survival outcomes and three immune subtypes. The prognostic differences between the three immune subtypes of LUAD were analyzed in relation to clinical data. There were statistically significant differences in survival between the three subtypes. The absolute value of slope of C3 is significantly higher than that of C2 and C1. Among the three subtypes, C1 had the worst prognosis, C2 had an intermediate prognosis, and C3 had a considerably better prognosis (follow-up was 10, 15, and 15 years for C1, C2, and C3, respectively; **Figure 3A**). Furthermore, Multivariate Cox regression analysis revealed that the cluster (HR: 0.681; 95% CI: 0.534-0.868; *P* < 0.002), pathological stage (HR: 2.374; 95% CI: 1.726-3.265; *P* < 0.001), T stage (HR: 2.208; 95% CI: 1.506-3.238; *P* < 0.001), and N stage (HR: 2.471; 95% CI: 1.831-3.335; *P* < 0.001) were all significant, indicating the cluster was an independent immune prognostic factor (**Figure 3B**).

### 3.4 Immune microenvironment of three subtypes

**Figure 4:**
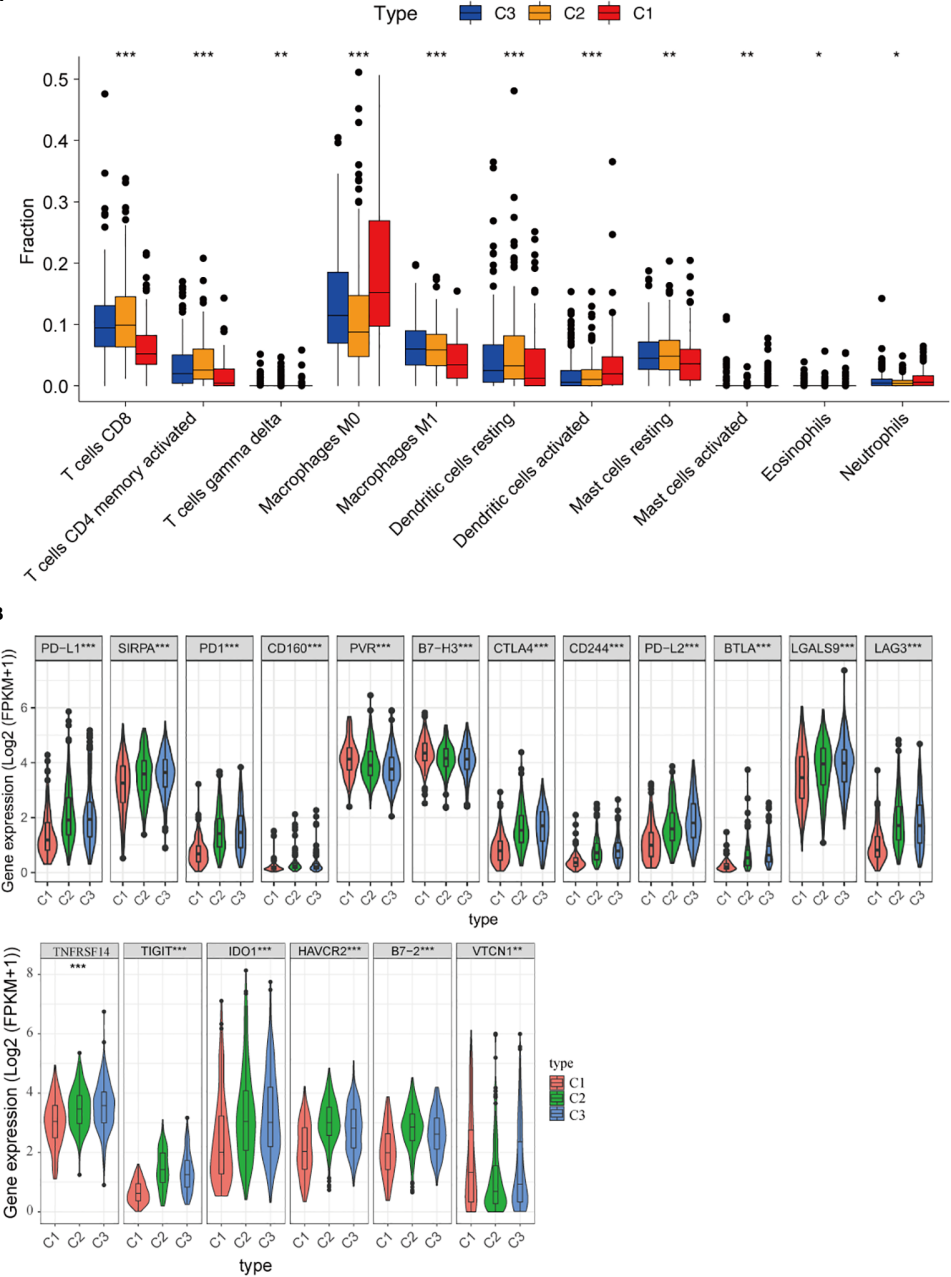
Immune microenvironment of three subtypes. **(A)** Comparison of the distribution of immune cells in three clusters; **(B)** The expression of immune checkpoint-related genes in three clusters. *, *P* < 0.05; **, *P* < 0.01 and ***, *P* < 0.001.

Next, the CIBERSORT algorithm was used to study the composition of TIICs between the subtypes and calculate the ratios of 22 immune cells in the microenvironment (41). We found that tumors in C1 were enriched with macrophages M0 cells, activated dendritic cells, and eosinophils, whereas tumors in C3 were enriched with CD8^+^ T cells, activated CD4^+^ memory T cells, and macrophages M1 cells (**Figure 4A**). Specific markers and functions of several immune cells are shown in **Supplementary Table 2**.

Besides, the associations between clusters and the expression of 18 different immune checkpoints were investigated, and we discovered that there were also significant differences in the expression of immune checkpoints in the three classifications, including immune checkpoints that are already in clinical use, such as PD-L1, PD1, CTLA4, LAG3, IDO1, HAVCR2, as well as promising targets for immunotherapy, such as CD160, PVR, B7-H3, SIRPA, CD244, B7-2, LGALS9, TIGIT, VTCN1 and NFRSF14 (**Figure 4B**). These findings suggested that “hot tumors” are more likely to occur in C3 patients, who were suitable for subsequent immunotherapy. Thus, the classification based on 29 immune signatures may provide a theoretical basis for immunotherapy of patients.

### 3.5 Gene mutational patterns of immune-based subtypes: Cluster 1 and Cluster 3

**Figure 5:**
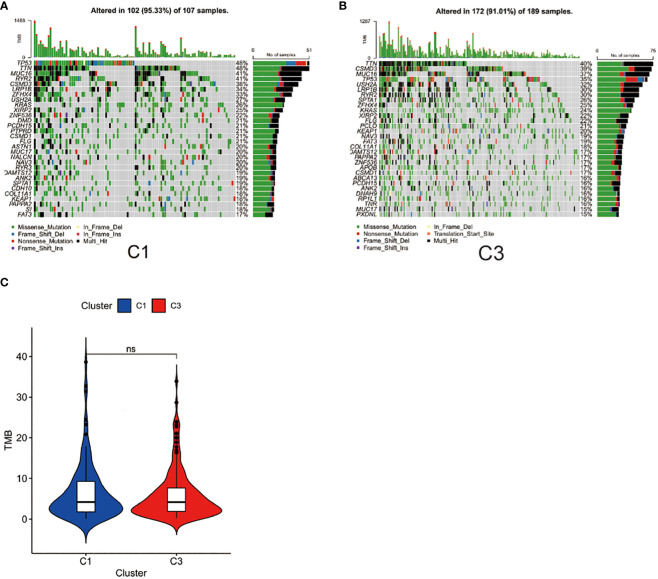
Gene mutational patterns of immune-based subtypes: Cluster 1 and Cluster 3. **(A)** Top 30 gene mutations in C1 subtype; **(B)** Top 30 gene mutations in C3; **(C)** Differences between the TMB scores of C1 and C3.

TMB is a biomarker that can help predict a patient’s response to immunotherapy (42). We demonstrated the key visualizations generated using maftools in TCGA cohorts. We identified 30 genes with frequent mutations in C1 of the TCGA cohort. The top 10 genes with the most frequent mutations were *TP53* (48%), *TTN* (48%), *MUC16* (41%), *RYR2* (41%), *CSMD3* (38%), *LRP1B* (34%), *ZFHX4* (33%), *USH2A* (27%), *KRAS* (26%), and *XIRP2* (25%) (**Figure 5A**). In C3 of the TCGA cohorts, the 10 most frequently mutated genes were *TTN* (40%), *CSMD3* (39%), *MUC16* (37%), *TP53* (35%), *USH2A* (32%), *LRP1B* (30%), RYR2 (30%), SPTAE (26%), *ZFHX4* (25%) and *KRAS* (24%) (**Figure 5B**). The TMB score was calculated using WES data from TCGA database. However, no significant differences were found between the TMB scores of C3 and C1 (**Figure 5C**).

### 3.6 Immune-related pathways associated with immune-based subtypes

**Figure 6:**
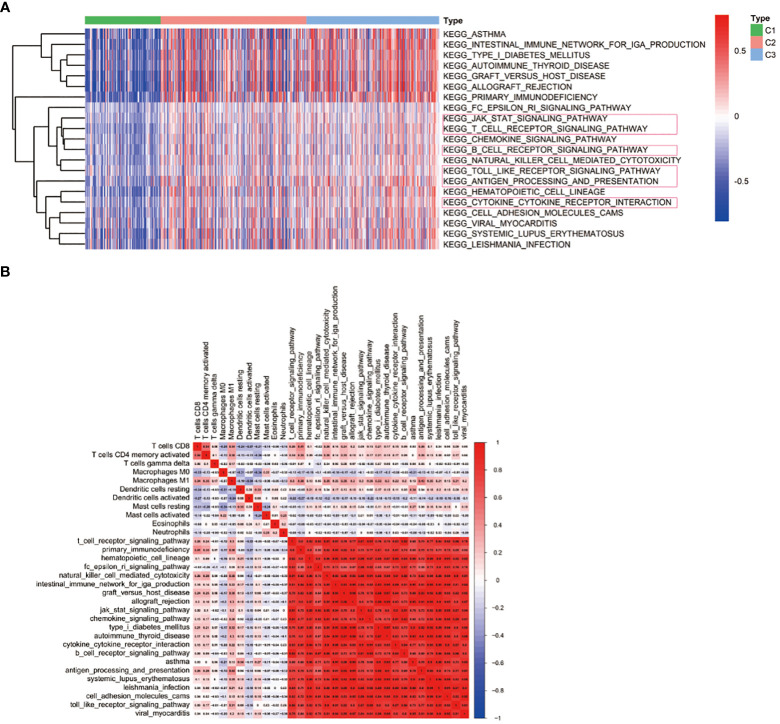
Immune-related pathways associated with immune-based subtypes. **(A)** KEGG pathway enrichment analysis; **(B)** Heat map displaying the association of immune cells and signaling pathways.

To better evaluate the connection between immune-related pathways and 29 immune signatures in LUAD, we performed GSEA analysis. We found the expression of 21 KEGG pathways were significantly upregulated in C3 and moderately upregulated in C2 while those pathways were inactivated in C1. GSEA analysis of the differentially enriched pathways of KEGG revealed that C3 had significantly activated immune-related pathways, such as the T cell receptor signaling pathway, the B cell receptor signaling pathway, the cytokine-cytokine receptor interaction, the JAK-STAT signaling pathway, the Toll-like receptor signaling pathway, and the antigen processing and presentation pathway (**Figure 6A**). These results further suggested that patients with C3 subtypes are more prone to hot tumors. What’s more, the correlation matrix of all 11 TIICs and 21 KEGG pathways in the TCGA cohort is depicted in **Figure 6B**. And we found that the CD8^+^ T cell and activated memory CD4^+^ T cells had a strong positive correlation with T cell receptor signaling pathways. Furthermore, M1 Macrophages also had a strong correlation with the T cell receptor signaling pathway, the antigen processing and presentation pathway, and the toll-like receptor signaling pathway.

### 3.7 Construction of the PPI network and identification of hub genes

**Figure 7:**
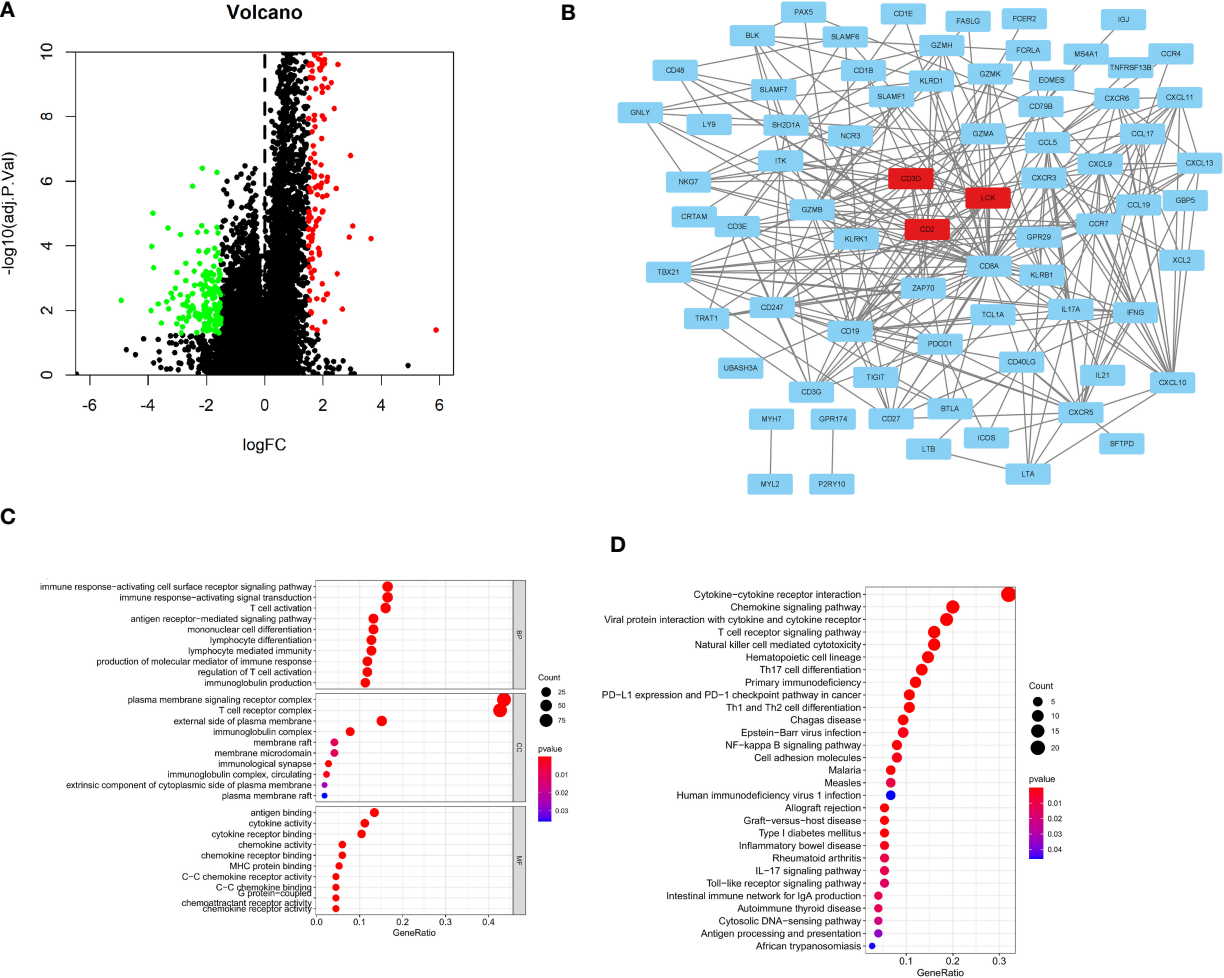
Construction of the PPI network and identification of Hub genes. **(A)**The volcano plots depicting immune-related DEGs; **(B)** PPI network displaying the association with 74 DEGs; **(C)** GO analysis showing enriched GO term; **(D)** KEGG pathway enrichment analysis.

To further determine specific indicators, differential gene expression analysis was performed. The volcano plot displays DEGs between C1 and C3 (**Figure 7A**). Further, the PPI network revealed interactions among these 74 DEGs (**Figure 7B** and **Supplementary Table 3**). We further analyzed the expression of 74 DEGs in both normal and tumor tissues. We found that 47 genes were upregulated in tumor tissues compared to normal tissues, such as CD48, TIGIT, GNLY, CCL19, CCL5, CXCL13, CCL17 and NKG7 (**Supplementary Figure 1**). GO analysis revealed that the DEGs between the C1 and C3 were enriched in immune response-activating cell surface receptors (BP), plasma membrane signaling receptor complexes (CC), and antigen binding (MF; **Figure 7C**). KEGG analysis revealed that DEGs were enriched in cytokine-cytokine receptor interaction, T cell receptor signaling pathway, natural killer cell-mediated cytotoxicity, PD-L1 expression, and PD-1 checkpoint pathway in cancer (**Figure 7D**).

## 4 Discussion

In the field of immuno-oncology, ICIs have revolutionized the treatment and management of cancer. However, only a small fraction of patients with LUAD demonstrate a sustained response to treatment. It is therefore crucial for clinicians to carefully select patients for anti-PD-1/PD-L1 treatment. Furthermore, finding a classification system based on 29 immune signatures to help screen LUAD patients who are more suitable for IO is critical. PD-L1 expression is being investigated as a potential predictor of response to anti-PD-1 therapy (11, 43, 44). There is a growing body of evidence that intratumoral T-cell infiltration, IFN signaling, or checkpoint molecules may facilitate a clinical response (45–47). Recent randomized trials revealed that combined-agent chemotherapy is superior to single-agent chemotherapy (48, 49). Therefore, we believe that, in comparison to individual immune parameters, the combination of multiple immune parameters has greater predictive power. Consequently, our study included a total of 29 immune signatures to analyze LUAD data in TCGA database. We found that patients in C3 had higher expression levels of CTLA4, PD-1, PD-L1, LAG3, IDO1, and HAVCR2. Combining the expression levels of these costimulatory molecules with lymphocyte subset analysis may allow for a novel method to assess immune dysfunction in patients with LUAD.

Proteomics (50), immunohistochemistry staining (51–53), and genetic characteristics (54) allow for more accurate cancer classification and prognosis prediction. However, these methods are primarily focused on tumor cells, while ignoring an important component of tumors known as the tumor immune microenvironment (TIME). Several factors, including lymphocyte density and phenotypes infiltrating the tumor, influence the immunotherapy response (55–58). The anti-tumor immune response of patients is crucial to their survival, giving rise to the now widely accepted concept of tumor immune contexture (56, 59, 60). However, cancer classification modalities continue to rely on traditional methods, not only for therapeutic purposes but also for classification purposes.

The 29 immune components included in this study could thoroughly cover and describe the immune status of tumors. Currently, immunotherapy appears to rely on eliciting an immune response from CD8+ T cells, which have a strong correlation with patient survival and direct tumor killing (61, 62). In addition to CD8+ T cells, our study includes some recent research hotspots, such as macrophages (63, 64) and dendritic cells (65), which have been shown to contribute to antitumor immunity and predict good prognoses in cancer patients. CAR-NK cells and adoptive transfer of NK cells are being investigated in both preclinical and clinical settings. NK cells have also been linked to the activation and inhibition of certain immune pathways (66, 67). Therefore, in addition to the aforementioned “star immune cells,” we included APC, Th1, Th2, IFN-γ, and other cells or cytokines.

In our study, we filtered and downloaded LUAD data from TCGA. Then, 29 immune signatures were obtained from the GSVA to assist in classifying patients into the three clusters (C1, C2, and C3) based on their scores in the aforementioned indicators. The correlation between 29 immune features and clinical features was further validated. We compared the prognostic outcomes for the three LUAD subtypes and found that C1 had the worst prognosis and C3 had the best prognosis. The composition of the tumor microenvironment was compared between subgroups, and C3 showed a significant increase in CD8+ T cells. Additionally, we conducted a GSEA analysis to investigate potential mechanisms underlying the immune response-related clustering of LUAD subgroups. It can help shed light on the combined strategy.

The aim of this study is to establish a new and comprehensive immune signature scoring system for LUAD patients. This study identified 3 multimarker-defined immune clusters.

At present, it is an urgent need that optimization of prognostic indictors should be considered for development of immune efficacy-related biomarkers toward clinical practice. We hope that this scoring system, combined with the clinical immunologic drug selection guidelines and the clinician’s experience, will optimize the process by which clinicians determine whether to treat patients with immunotherapy and reduce the unnecessary burden of immunologic drugs on patients. And most importantly, we hope to contribute to a better treatment of cancer patients.

Due to the complexity of predicting response to ICI treatment in LUAD, in addition to immune-related components, histopathology, imaging, and certain clinical factors should also be considered. Both pathological factors and tumor spread rate influence the response to anti-PD-1/PD-L1, necessitating further research. An integrative multi-parameter approach is a novel strategy in this regard. Furthermore, this classification should be investigated further in patients receiving IO to determine its predictive ability. In addition, a thorough understanding of the molecular mechanisms underlying LUAD tumor immunogenicity is required. In the future, the advent of immunotherapy has improved patient survival outcomes in LUAD due to the biomarker-based approach, and parallel research would be conducted on various biomarkers.

Research into the immunological characterization of LUAD tumors and its utilization to optimize immune-based therapy is in its early stages. Therefore, the immune classification of LUAD is crucial. By analyzing the immunologic profile and interaction of LUAD with the tumor microenvironment, this study sheds light on the immunology of this tumor.

## Data availability statement

The datasets presented in this study can be found in online repositories. The names of the repository/repositories and accession number(s) can be found in the article/**Supplementary Material**.

## Author contributions

Conception and design: XZ and BC. Acquisition of data: XZ. Analysis and interpretation of data: XZ, DJ, XYZ, and WZ. Writing and review of the manuscript: XZ and SL. Revision of the manuscript and study supervision: BC. All authors contributed to the article and approved the submitted version.

## Supplementary material

Supplementary Figure 1 The expression of 74 Hub genes in lung tumor and normal tissue.
