# Supplementary figures and images for: A signature-based classification of lung adenocarcinoma that stratifies tumor immunity
Source: Front Oncol. 2023 Jan 12;12:1023833. doi: 10.3389/fonc.2022.1023833 (PMC9878554; doi:10.3389/fonc.2022.1023833)

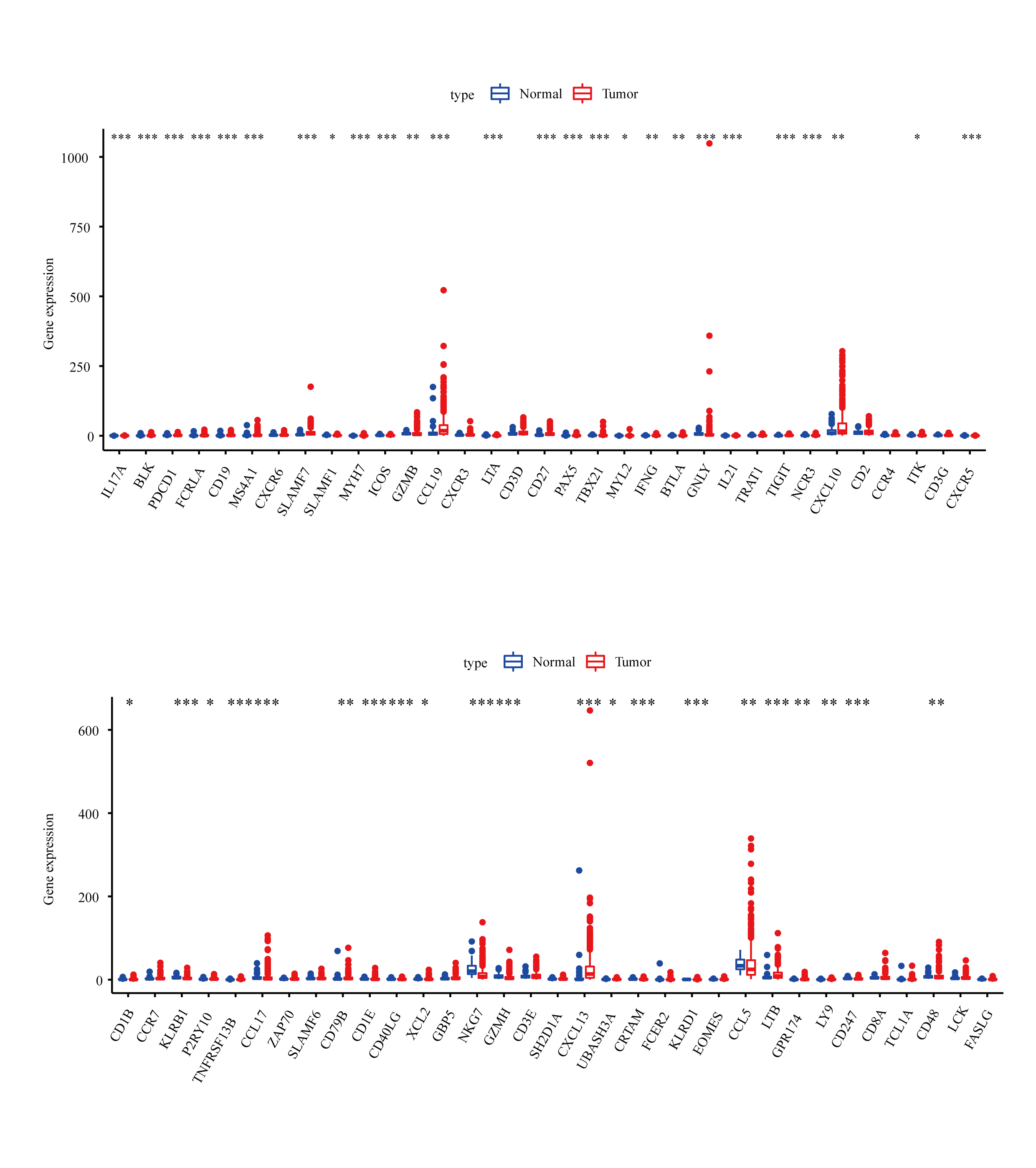

Supplement: Supplementary Figure 1 — The expression of 74 Hub genes in lung tumor and normal tissue. [file Image_1.tif]
